# A mixed studies systematic review of the unmet treatment, support, and information needs of cancer survivors in managing menopause symptoms

**DOI:** 10.1007/s00520-026-11002-4

**Published:** 2026-07-23

**Authors:** Dorcas Serwaa, Shanton Chang, Khadijeh Onsory, Nipuni Susanto, Martha Hickey, Michael Jefford, Michelle Peate

**Affiliations:** 1 Department of Obstetrics, Gynaecology, and Newborn Health, Faculty of Medicine, Dentistry and Health Sciences, The University of Melbourne, Melbourne, Australia; 2 Department of Obstetrics, Gynaecology, and Newborn Health, Faculty of Medicine, Dentistry and Health Sciences, The Royal Women’s Hospital, Melbourne, Australia; 3 School of Computing and Information Systems, The University of Melbourne, Melbourne, Australia; 4 Centre for Health Services Research in Cancer, Department of Medical Oncology and the Australian Cancer Survivorship Centre, Peter MacCallum Cancer Centre, Melbourne, Australia; 5 Sir Peter MacCallum, Department of Oncology, The University of Melbourne, Melbourne, Australia

**Keywords:** Menopause, Cancer survivors, Treatment needs, Information needs, Support needs, Systematic review

## Abstract

**Introduction:**

Menopausal symptoms are common after cancer treatment, with wide-ranging impacts on cancer survivors’ daily living. While evidence shows that cancer survivors have menopause-related needs, these have not been synthesised. This systematic review aims to identify cancer survivors’ unmet needs in managing menopausal symptoms.

**Method:**

The study was registered with the Prospective Register for Systematic Reviews (CRD42024464668). We searched four databases (MEDLINE, Embase, PsycINFO, CINAHL) for original qualitative or quantitative research published in English between 2013 and 2025, and focusing on cancer survivors’ unmet menopause-related needs. Manual searches of reference lists were also conducted. Quantitative studies were reported descriptively, while qualitative data was analysed using inductive thematic analysis.

**Results:**

The search returned 8256 abstracts, of which, 20 studies (13 qualitative and 7 quantitative) met the inclusion criteria. Three key themes were identified regarding unmet needs: treatment, support, and information. Treatment needs involved survivors untreated, those finding treatment ineffective, and desire for more options. Between 26 and 80% were untreated or self-managing their symptoms, 15–76% found their treatments ineffective, and 23–68% desired additional treatment. Support needs included inadequate support from healthcare professional. Some felt abandoned, their symptoms dismissed, and therefore needed extra support in managing their symptoms. Gaps in access to information about cancer treatment-induced menopause and its management were identified. Most sought information from diverse sources; however, their needs were not fully met.

**Conclusion:**

The review revealed unmet needs in treatment, support, and information for cancer survivors managing menopausal symptoms. These findings could guide providers to improve practice and inform supportive care policies.

## Introduction

Cancer is a major public health burden [1, 2], and about 60% of all cancer cases and mortality are in women [2, 3]. With early detection, advances in treatment, and improved clinical and social support systems, survival rates have improved significantly over decades, globally [2, 4]. This translates to more women living with treatment effects [5, 6]. While some side effects might resolve naturally over time, others may persist over a period [7, 8].

Menopause symptoms, such as vasomotor (hot flushes, night sweats), and genitourinary symptoms, are some commonly reported long-term consequences after cancer [9, 10], attributable to the ovarian toxicity of hormonal therapies, chemotherapy, radiotherapy, and from surgical removal of the ovaries [11]. These symptoms, if unmanaged, can negatively impact sleep, intimacy, and the quality of life (QoL) of cancer survivors [8, 12]. While there are several effective, evidence-based interventions available for managing these symptoms [13], menopause management continues to be a leading unmet need in cancer survivors [14, 15].

Recent studies show that recognising and responding to the needs of cancer survivors result in improved health outcomes, overall well-being, and satisfaction with care received [16, 17]. Although existing evidence shows numerous menopause-related needs of cancer survivors, there is currently no systematic review that synthesises these needs to highlight gaps in care. Most cancer survivorship reviews to date have primarily focused on broader cancer-related unmet needs, such as treatment concerns, fear of recurrence, and psychological distress, neglecting menopause-related unmet needs [18–24]. Likewise, literature reviews among cancer survivors with menopausal symptoms have predominantly examined the options, the efficacy and/or safety of interventions aimed at managing menopausal symptoms [25–30]. While these reviews provide evidence to guide clinical decision-making, they offer limited understanding into the real-world experiences and unmet needs of  cancer survivors managing their symptoms.

Therefore, the aim of this systematic review was to identify and synthesise evidence on the unmet needs of cancer survivors in managing their menopausal symptoms. The findings from this review will offer valuable insights to healthcare providers and policymakers seeking to develop new services or adapt existing ones, to better address the unique unmet need reported by cancer survivors in managing their menopausal symptoms.

## Methods

### Design

**Fig. 1:**
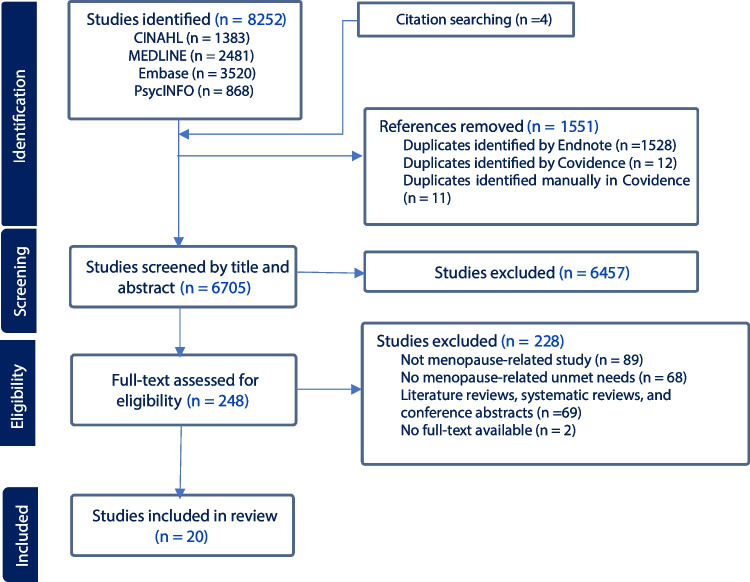
PRISMA flowchart of the literature search and screening process

This systematic review employed mixed-method approach. A mixed-method systematic review (MMSR) was used to synthesise and integrate evidence from qualitative and quantitative studies in a structured manner [31]. MMSR enable evidence triangulation, offering deeper insights into phenomena and revealing agreements or disparities across the available studies [31]. MMSR are increasingly common in cancer survivorship research, as demonstrated by recent studies [32–34]. This study adopted this method to generate conclusions relevant for policy and practice. The review followed Preferred Reporting Items for Systematic Review and Meta-Analysis (PRISMA) guidelines [35] (Fig. 1). It was registered with Prospective Register for Systematic Reviews (CRD42024464668).

### Definitions

- The World Cancer Research Fund defines cancer survivors as “individuals who have been diagnosed with cancer, including before, during and after treatment” [36].
- Menopause, according to the World Health Organisation, is marked by the end of monthly menstruation, also known as a menstrual period. Symptoms include hot flushes, night sweats, dryness, and itching of the vulva/vagina [37].
- Unmet need in this context is defined as a requirement for action or resources that is necessary, desirable, or useful [38], for cancer survivors to manage menopausal symptoms and optimise well-being. We included studies that (a) explicitly used the term “unmet need(s)”; (b) reported participants-expressed needs that remained unmet, even if not labelled as such; and (c) described finding from which unmet need could be reasonably inferred.

### Search strategy

Two strategies were employed: four electronic databases (Ovid MEDLINE(R) ALL, Embase Classic + Embase, APA PsycINFO, and CINAHL) were searched using MeSH terms and keywords (Appendix 1). The search was limited to females, English language, and human studies from 2013 to 2025. As cancer treatments change over time, so do the side effects, including menopause; hence, the search was restricted to 13 years for relevance. Manual searches of reference lists were also conducted. The initial search was performed on 13th October 2023 and updated on 12th March 2025.

### Search selection

Search results (*n* = 8256) were exported to EndNote (version 20) [39] to facilitate the removal of duplicates (*N* = 1528), then to Covidence systematic review software [40], for further deduplication (*n* = 23), title and abstract screening. Study inclusion criteria were that it was (a) carried out in cancer survivors experiencing menopause symptoms; (b) an original research article; (c) reported menopause-related unmet needs; (d) in English; and (e) published from 2013 to 2025. The exclusion criteria were studies that (a) reported on menopausal symptoms in the non-cancer survivors; (b) reported no menopause-related unmet needs; (e) non-original articles, and conference abstracts. Titles and abstracts were independently screened for inclusion by two authors (DS and NS). DS and KhO independently conducted full-text screening. Discrepancies were discussed (DS and NS or KhO) and consensus achieved.

### Data extraction

Data was extracted into an Excel table informed by COnsolidated criteria for REporting Qualitative research (COREQ) [41] and the STtrengthening the Reports of OBbservational studies in Epidemiology (STROBE) [42] guidelines. Extracted information included publication details (author name, etc.), study characteristics (recruitment setting, study design, etc.) and participants (age, cancer type), and key outcomes relating to menopause-related unmet needs (Table 1). DS and KhO independently extracted the data and resolved discrepancies. MP and SC reviewed and agreed on the thematic structure appropriate for reporting the findings.

**Table 1:** Characteristics of included quantitative studies (*n* = 7)

Author, year published	Study aim^a^	Country	Recruitment setting	Study design	Population criteria	Sampling strategy and analyses	*n*	Age in years; mean (SD, range) or median [IQR]	Relevant findings^b^
Cole et al. 2022 [60]	To (1) identify which vasomotor symptoms are most bothersome for patients; (2) identify a patient-derived definition of optimal control of vasomotor symptoms; and (3) determine the perceived efficacy of previous interventions for vasomotor symptoms based on aim 2 endpoints	Canada	Recruited from routine clinical visit to oncology centre and database of two tertiary cancer centres	Cross-sectional survey	Women ≥ 18 years with early breast cancer (stages I–III) and VMS	Convenience sampling. Descriptive statistics	357	58 [23–83]	• 80% had not received any treatments or healthcare professionals’ recommendations for vasomotor symptoms • 68% interested in using an intervention
Capelan et al. 2017 [15]	To (1) estimate the prevalence of unmet needs in breast cancer survivors entering the Open Access Follow-Up programme; (2) describe the nature of the unmet needs, and what factors might identify those patients at the highest risk of having unmet needs at completion of hospital-based treatment	UK	Recruited through a specialist cancer hospital	Cross-sectional survey	Women with a previous diagnosis of early (stages I–III) breast cancer who had completed their initial treatment (surgery, chemotherapy, radiotherapy)	Convenience sampling. Descriptive and inferential statistics	625	59 [27–97]	• 23% expressed unmet need for management of hot flushes
Fenlon et al. 2022 [46]	To investigate the perceptions amongst patients and healthcare professionals of the level of unmet need relating to hot flushes and night sweats in women with breast cancer, and current management practices	UK	Recruited through online discussion forums on Breast Cancer Care website and Twitter account	Online cross-sectional survey	Breast cancer survivors	Convenience sampling. Descriptive statistics	665	50 [25–69]	• 34% were not asked about vasomotor symptoms • 57% not offered any drug treatment • 1.8% had been offered cognitive behavioural therapy • 18% had been offered acupuncture • 76% on ineffective medical treatments • 35% found cognitive behavioural therapy unhelpful • 49.2% found acupuncture unhelpful
Miyashita et al. 2015 [62]	To identify unmet information needs and examine the relationships between unmet information needs and quality of life in young breast cancer survivors	Japan	Recruited through two tertiary institutions	Cross-sectional survey	Japanese women diagnosed with breast cancer ≤ 45 years	Consecutive sampling. Descriptive and inferential statistics	163	39.9 (4.5, 18–45) and 44.8 (6.6, 21–64)	• 27% received no information about secondary menopause associated with hormonal therapy • 32.5% received no information about secondary menopause associated with chemotherapy • 18.5% were unsatisfied with hormonal therapy-related menopause information • 24.5% were unsatisfied with chemotherapy-related information
Palmer et al. 2016 [59]	To examine the unmet needs for symptom management and the relationships between unmet needs, symptom burden, and quality of life	USA	Recruited through a tertiary cancer centre	Cross-sectional survey	Completed initial/primary breast cancer therapy within 12 months prior, ≥ 18 years, confirmed diagnosis of ductal carcinoma in situ or nonmetastatic breast cancer, scheduled for a follow-up visit	Consecutive sampling. Descriptive and inferential statistics	164	55.45 (11.97)	• 26% of 73% experiencing hot flushes expressed need for intervention • 27% of the 63% who experienced night sweat expressed unmet needs • 28% of the 37% experiencing pain during sex had unmet need
Peate et al. 2020 [14]	To determine the nature and severity of vasomotor symptoms, sexual problems, mood, and sleep disturbance in community-dwelling breast cancer patients, whether and where they received treatment for these symptoms and their satisfaction with treatment received	Australia	Recruited through online survey sent to members of Breast Cancer Network Australia	Online, cross-sectional survey	Breast cancer patients	Convenience sampling. Descriptive and inferential statistics	524	55.21 (8.46, 30–89)	• 32% offered treatment for symptoms • Treatment effective for 17%, “somewhat effective” for 49% • 25.9% self-managing symptoms or had no help • 70% self-managing sexual problems • 60% and 53% of participants with menopausal symptoms and sexual problems respectively wanted more support for symptom management
Benedict et al.2016 [53]	To (1) describe survivors’ unmet information needs regarding fertility topics, their reproductive concerns, and the degree of decisional conflict they experienced when prompted to consider the decision to pursue fertility preservation in the future; (2) identify potential factors contributing to decision-making distress, the extent to which unmet information needs and reproductive concerns were related to decisional conflict about future fertility preservation was also evaluated	USA	Recruited through cancer centre, survivor advocacy groups, social media, and e-mail listservs	Online cross-sectional survey	Female survivors aged 18–35 years, successfully completed treatment at least 1 year before study	Convenience sampling. Descriptive statistics	346	29.9 (4.1, 18–35)	• 60% did not receive information about their risk of early menopause

^a^This column presents the primary aim of the included studies

^b^Relevant findings: This column presents only the results pertinent to our current review’s objectives, rather than a summary of each individual study’s findings

### Quality assessment

The Joanna Briggs Institute (JBI) checklists for qualitative (Appendix 2) and quantitative (Appendix 3) studies were used for critical appraisal; scores ≤ 49%, 50–69%, and ≥ 70% “yes” were considered low, moderate, and high quality, respectively [43]. No studies were excluded based on quality, as all provided relevant data on cancer survivors'  experiences. However, during the data synthesis and interpretation, studies rated as high‑quality studies were prioritised when forming core themes, and moderate-quality studies were used to support or refine those themes, but interpreted with caution. Where findings from high- and moderate-quality studies were in discordance, greater weight was placed on the evidence from the high‑quality studies. DS and KhO independently conducted the quality appraisals and resolved discrepancies by consensus.

### Data analysis and synthesis

For this MMSR, a sequential explanatory method guided the synthesis and analysis of the identified studies [32]. With this approach, synthesis of the quantitative studies preceded and informed the later synthesis of the qualitative studies. Where possible, both studies were triangulated, such that either the qualitative findings expanded on the quantitative data or vice versa. This approach to allowed for a more nuanced interpretation.

For all quantitative studies, we extracted data on cancer survivor's unmet needs in managing menopausal symptoms, including those directly reported (i.e. unmet need) by the included studies or as determined (i.e. implied need) by the current study authors, based on the definition for unmet need [38]. The quantitative data were limited and the population characteristics were variable making meta-analysis inappropriate. Therefore, the findings were reported descriptively.

For the qualitative data, we extracted direct quotes and/or the primary or current researchers’ interpretation of unmet needs verbatim from each study. Each extract was treated as single transcript and imported to Nvivo V. 12 [44], for analysis. We conducted inductive thematic analysis as outlined by Braun and Clark [45]. This involved reading and re-reading of data, and initial line-by-line coding to identify subthemes. Subthemes were refined and grouped into overreaching themes. DS led the coding process, developed themes, and consulted with MP and SC to ensure accuracy and reliability, and to address reflexivity.

## Results

### Identification of included studies

The search produced 8256 references. After de-duplicates, 6705 titles and abstracts were screened, and 6457 papers excluded based on wrong population, outcome, and non-original research. Of 248 full-text reviewed, 20 papers were included (Fig. 1).

### Characteristics of included studies

Twenty studies were included (Tables 1 and 2): eight primarily investigated cancer survivors’ menopausal symptoms and/or unmet needs [4, 14, 46–51], while other reported them as secondary findings [15, 52–59], or implied need–determined either by the primary [60, 61], or current study authors [62]. Studies were conducted in nine countries, mostly USA (*n* = 6) [53, 55–57, 59, 61], UK (*n* = 4) [4, 15, 46, 59], and Australia (*n* = 3) [14, 50, 63]. Total sample was 3176, with 2844 (range 163–665) from the quantitative and 332 (range 10–58) from the qualitative studies. The participants aged 18–97 years. Eleven studies focused on post-treatment cancer survivors [15, 47–49, 51, 53–57, 59], two on active treatment [50, 61], and seven included both groups [4, 14, 52, 58, 62, 64, 65]. Seventeen studies were among breast cancer survivors and one each on gynaecological [63], and gestational trophoblastic [49], and one on survivors of diverse cancer types [53].

**Table 2:** Characteristics of included qualitative studies (*n* = 13)

Author, year published	Study aim^a^	Country	Recruitment setting	Study design	Population criteria	Sampling strategy and analyses	*n*	Age in years; mean (SD, range) or range or median [IQR]	Relevant findings^b^
Cruickshank and Hume 2014 [4]	To describe experiences and expectations of both women with cancer and the healthcare professional who care for them in relation to the management of vasomotor and genitourinary symptoms of menopause	UK	Recruited through outpatient department of a tertiary cancer centre	Phenomenological focus group and interview study	≥ 18 years, female, diagnosed with primary breast cancer, described experiencing menopausal symptoms to the healthcare professionals	Purposive sampling. Thematic analysis	14	40–70	• Lack of baseline assessment of reported menopausal symptoms • Desire for baseline and follow-up assessment of menopausal symptoms • Inadequate information about cancer treatment and menopause • Lack of support from healthcare professionals • Relied on diverse sources for information and support • Expressed desire for more support
Balneaves et al. 2016 [51]	To identify the complementary therapy and related information to be included in a decision aid (called MyChoices™) for breast cancer survivors experiencing menopausal symptoms	Canada	Recruited through tertiary institution, and support groups	Phenomenological focus group and interview study	Women diagnosed with breast cancer (stages I–IIIb) in last 5 years without recurrence, completion of primary cancer treatment, and considering complementary therapies for menopausal symptom management	Purposive sampling. Thematic analysis	22	30–50 +	• Need for information about menopause before cancer treatment • Need for information about symptom management • A lack of support from healthcare professionals • Desire for more support
Johnsson et al. 2023 [47]	To explore the extent to which women treated with adjuvant endocrine therapy perceived healthcare professionals addressed the menopausal side effects which they experienced	Sweden	Recruited through three tertiary and one primary care	Phenomenological focus group and interview study	Women with a hormone receptor-positive breast cancer who had an ongoing adjuvant endocrine therapy started within the past 7 years	Purposive sampling. Content analysis	58	36–78	• Lack of relevant information about symptom management • Relied on different sources for information • Concerns about inconsistencies in information received • Need for support
Sousa et al. 2017 [50]	To explore knowledge, attitudes, and experiences of genitourinary symptoms in women receiving endocrine therapy	Australia	Patient database of two tertiary hospitals	Phenomenological interview study	Woman diagnosed with early breast cancer (stages IA, IB, IIA, and IIB), ≥ 18 years, and receiving adjuvant endocrine therapy (either tamoxifen, or an aromatase inhibitor)	Purposive sampling. Thematic analysis	19	42–77	• Difficulties in accessing information about genitourinary symptoms • Symptom dismissal by healthcare professionals • Inadequate support from healthcare professional • Difficulty in assessing the credibility of information obtained from diverse sources • Need for information about symptom management • Desire for more support
Limbacher et al. 2022 [61]	To describe how menopausal symptoms are presented, discussed, and resolved in the clinical encounters during early-stage chemotherapy between women with breast cancer and their healthcare professionals	USA	Recruited through seven tertiary institutions	Non-participatory observational (consultation recordings) [secondary data]	Women with stage I–III breast cancer during chemotherapy	Purposive sampling. Thematic analysis	37	52 (from parent study)	• The lack of support from healthcare professionals (symptoms dismissal and lack of empathy from healthcare professionals)
Black et al. 2020 [57]	To determine the concordances and discordances in pre-menopausal breast cancer survivors’ identified sexual and reproductive health needs	USA	Recruited through community-based approach and cancer centre-based breast cancer support group meetings, cancer centre–based listservs for young breast cancer survivors, and referrals from the advisory committee	Phenomenology, grounded theory, narrative interviews, and case study	Women with a history of breast cancer, diagnosed between 18 and 45 years, and living in North Carolina	Convenience sampling. Thematic analysis	17	45.8 (7.2,37–64)	• Inadequate information prior to cancer treatment • Challenges in finding treatment for genitourinary symptoms • Diverse sources for information • Concerns about the credibility of information received • Desired solutions to address sexual-related symptoms
Sparidaens et al. 2022 [48]	To identify the fertility and early menopause-related information needs of young breast cancer survivors and to design, develop, and implement online information material with stakeholder input	The Netherlands	Recruited through a large teaching hospital	Phenomenological interview study	Young female breast cancer survivors, 20–45 years, who had completed their initial treatment (i.e. surgery, chemotherapy, and/or radiation therapy), and were currently in their follow-up period	Convenience sampling. Thematic analysis	18	35.5 (21–44)	• Need for information about menopause before cancer treatment • Felt abandoned after cancer treatment • Depended on different sources for information Need for information about symptom management
Lopez et al. 2019 [58]	To explore the shared and unique supportive care needs of younger and older gynecological cancer patients and survivors across the care trajectory	Australia	Recruited through three tertiary hospitals	Phenomenological interview study	Women aged ≥ 18 years, 3 months–5 years post-diagnosis	Purposive sampling. Thematic analysis	29	27–70	• Want more information about coping with and overcoming sexual difficulties
Moreno et al. 2023 [56]	To characterise supportive care needs among women living with metastatic breast cancer	USA	Recruited through consent to contact lists, support groups, and community-based organisation	Phenomenological focus-group discussion	Women aged ≥ 18 years, diagnosed with stage IV (M1) breast cancer, can speak and read in English	Purposive sampling. Thematic analysis approach	22	60.3 (12.21, 34–84)	• Interest in symptom management strategies • Desire for more support on sexual-related symptoms • Inadequate information about treatment option • Desire for open, compassionate communication with their healthcare providers
Khajoei et al. 2024 [54]	To investigate the needs and experiences of breast cancer survivors	Iran	Recruited through university hospital	Phenomenological, interview study	Breast cancer survivors, 18–60 years, literacy, completion of treatment, non-metastatic cancer, and stable clinical conditions	Purposive sampling. Framework analysis	14	____	• Lack of information about treatment side effect • Not provided with training on how to manage their hot flushes
Ko et al. 2023 [55]	To understand the needs and experiences of Black women after they have completed active breast cancer treatment and their own specific needs and concerns during breast cancer survivorship	USA	Recruited through physicians and cancer registry data	Phenomenological, focus groups and cross-sectional surveys	Self‐identification as Black or African American; aged 18–74 years, diagnosed with invasive breast cancer in the past 5 years, and speak English, Spanish, or Haitian Creole	Convenience sampling. Descriptive and framework analysis	43	54 (IQR 15)	• Felt hot flushes symptom were dismissed • Expressed the need for anticipatory information regarding hot flushes as cancer treatment side effects
Singh et al. 2024 [49]	To (1) explore patient’s experience of chemotherapy-induced menopausal symptoms; (2) to ascertain how patients tried to alleviate their symptoms and how health professionals supported them in order to identify current unmet needs	UK	Recruited through gestational trophoblastic neoplasia database in a hospital	Phenomenological, retrospective cross-sectional exploratory study using Interview and surveys	Women who had gestational trophoblastic neoplasia received multi-agent chemotherapy, completed treatment less than 2 years ago, evidence of temporary or permanent ovarian failure with raised follicle-stimulating hormone and luteinizing hormone in post-menopausal range, and able to provide informed consent	Purposive sampling. Descriptive and framework analysis	10	30–49	• Difficulty in between chemotherapy side effect and menopausal symptoms • Accessing support for menopausal symptoms • Need to consider timing of information about menopause
Villarreal‐Garza et al. 2019 [52]	To describe clinical and information needs, to identify unmet support services and to guide the further development of educational and supportive interventions for young breast cancer patients in Mexico	Mexico	Recruited from a prospective cohort information database	Phenomenological, an exploratory, cross‐sectional, qualitative study using focus group discussion	Patients with initial breast cancer, diagnosed within 6–12 months prior to study, ≤ 40 years at diagnosis and literate	Convenience sampling. Framework approach with interpretative description methodology	29	39 (29–44)	• Inadequate information about chemotherapy-induced menopause • Inadequate information about the symptoms of menopause

^a^This column presents the primary aim of the included studies

^b^Relevant findings: This column presents only the results pertinent to our current review’s objectives, rather than a summary of each individual study’s findings

Seven were quantitative studies, all used convenience sampling techniques [14, 15, 46, 53, 59, 62, 64], and four used face-to-face cross-sectional survey [15, 59, 60, 62]. All used questionnaire and applied both descriptive and inferential analysis [14, 15, 53, 59, 62] (Table 1). Three were high quality [14, 15, 60], and four moderate quality [46, 53, 59, 62] (Appendix 2). Thirteen studies were qualitative, with most using purposive sampling (*n* = 8) [45–49, 52, 56, 59] and phenomenological approaches (*n* = 12) [4, 45–50, 52–56]. Data were collected using interviews [4, 48, 52, 56], interview and focus group [45, 46, 49], or either with other methods [47, 50, 53, 55]. Eight studies used thematic analysis [4, 45, 48, 49, 54–56, 59] (Table 2), and all were high quality (Appendix 3). One study [15] used holistic needs assessment (HNI) to assess unmet needs, the reminder used researcher-designed questionnaire.

### Summary of findings

Three themes, namely, treatment, support, and information needs, were identified (Table 3), with eight subthemes. “Treatment needs” described whether the cancer survivors received treatment for their menopausal symptoms, found the treatment effective, or desired more treatment options. “Support needs” involved inadequate support from healthcare professionals (HCPs) and desire for further support. “Information needs” covered inadequate information about menopause pre-cancer treatment, insufficient guidance about symptom management, and the reliance on diverse information source—the range and reliability of sources used to obtain information about menopause and its management.

**Table 3:** Descriptive and analytic themes of unmet needs as identified in the included studies (*n* = 20)

Analytic themes	Descriptive themes	Quantitative studies	Qualitative studies
Treatment needs	Received no treatment for menopausal symptoms	Peate et al. [14]; Fenlon et al. [46]; Cole et al. [60]	—
	Found treatment ineffective	Peate et al. [14]; Fenlon et al. [46]	—
	A desire for more treatment	Capelan et al. [15]; Palmer et al. [59]; Cole et al. [60]	Moreno et al. [56]
Support needs	Inadequate support from healthcare professionals	Fenlon et al. [46]; Cole et al. [60]	Cruickshank and Hume [4]; Johnsson et al. [47]; Sparidaens et al. [48]; Sousa et al. [50]; Balneaves et al. [51]; Ko et al. [55]; Black et al. [57]; Limbacher et al. [61]
	Expression of a desire for more support	Peate et al. [14]	Cruickshank and Hume [4]; Johnsson et al. [47]; Singh et al. [49]; Sousa et al. [50]; Balneaves et al. [51]; Moreno et al. [56]
Information needs	Inadequate information about menopause pre-cancer treatment	Benedict et al. [53]; Miyashita et al. [62]	Cruickshank and Hume [4]; Johnsson et al. [47]; Sparidaens et al. [48]; Singh et al. [49]; Sousa et al. [50]; Balneaves et al. [51]; Villarreal‐Garza et al. [52]; Khajoei et al. [54]; Ko et al. [55]; Moreno et al. [56]; Black et al. [57]; Lopez et al. [58]
	Insufficient guidance on menopausal symptom management	—	Sparidaens et al. [48]; Sousa et al. [50]; Balneaves et al. [51]; Khajoei et al. [54]; Moreno et al. [56]; Black et al. [57]
	Reliance on diverse information source	—	Cruickshank and Hume [4]; Johnsson et al. [47]; Sparidaens et al. [48]; Singh et al. [49]; Sousa et al. [50]; Balneaves et al. [51]; Black et al. [57]

#### Theme 1: Treatment needs

Three studies reported on women receiving treatment for their menopausal symptoms [14, 46, 60], two on those who found their treatment ineffective [14, 46], and four on desire for treatment [15, 56, 59, 60] (Table 3).

##### Women who received no treatment

While three quantitative studies (1546 participants) explored the experiences of women with breast cancer whose menopausal symptoms remained untreated [14, 46, 60], no qualitative studies addressed this issue. Cole et al. [60] examined persons with early-stage breast cancer (stages I–III) recruited from oncology clinics at two tertiary cancer centres, finding that 80% received no formal treatment or recommendations for symptom management [60]. Peate et al. [14] reported 26% of a community sample of women with a history of breast cancer experienced vasomotor symptoms, sexual dysfunction, and other menopause-related issues who were either self-managing or receiving no support; among those with sexual-related problems, 70% were self-managing. Similarly, Fenlon et al. [46] found that 57% women had not been offered pharmacological treatment (e.g. venlafaxine, gabapentin, clonidine) for vasomotor symptoms. Few were offered evidence-based complementary therapies such as cognitive behavioural therapy (CBT) (1.8%) and acupuncture (18%) [46].

##### Women reporting ineffective intervention

Amongst women who received treatment for vasomotor symptoms, 76% reported that medical treatments were ineffective, while 35% found CBT and 49.2% found acupuncture unhelpful [46]. In the Peate et al. [14] study, which examined a broad range of menopausal symptoms, 34% of participants found interventions to be completely ineffective, 49% rated them “somewhat effective”, and only 17% considered them effective. Dissatisfaction with care was widespread, with 70% dissatisfied with management of menopausal symptoms and 87% dissatisfied with care for sexual symptoms. Cole et al. [60] found that 83% of women referred to dedicated menopause clinics reported beneficial outcomes. No qualitative studies explored the perceived effectiveness of treatments for menopausal symptoms.

##### Women with desire for more treatment

Three studies depicted women’s need for improved interventions for menopausal symptoms [15, 59, 60]. Capelan et al. [15] found that 23% of 625 women with stage I–III breast cancer in a specialist hospital reported unmet menopause treatment support need. Between 26 and 68% needed more support for vasomotor symptoms [59, 60]; 3% wanting a medical option, and 21% open to any treatment [60]. For genitourinary symptoms, 27–37% sought additional treatment for issues such as vaginal dryness (37%), decreased sexual interest (27%), and pain during sex (28%) [59]. These findings are supported by a qualitative study where cancer survivors with genitourinary symptoms reported a desire for more strategies to manage their symptoms [56].

#### Theme 2: Support needs

Twelve studies identified cancer survivors’ needs for support in managing their symptoms. Two quantitative [46, 60] and eight qualitative [4, 47, 48, 50, 51, 55, 57, 61] studies identified areas professional support could be strengthened. One quantitative [14] and six qualitative [4, 47, 49–51, 56] studies reported survivors’ desires for more support (Table 3).

##### Inadequate support from HCPs

Several studies presented findings that could help HCPs to enhance their support for cancer survivors with menopausal symptoms. While Cole et al. [60] found that 58% of 357 women with early-stage breast cancer were routinely asked about their hot flushes and Fenlon et al. [46] reported 66% of 665 breast cancer survivors receiving such inquiries, there remained 42% and 34% of survivors, respectively, who could have benefited but reported not being asked. In addition, qualitative studies [4, 47, 48, 50, 51, 61] provided insight into cancer survivors’ experiences with the support they received. Some felt abandoned post-cancer treatment [4, 48] and believed ongoing support, clearer guidance on effective menopausal symptoms management strategies could have prevented these feelings.

...I think you do have that feeling of being thrown out after you complete your treatment, … then you’ve got the menopause on top of that,…. (Cruickshank and Hume [4], patient 9)

Women who actively sought support from HCPs hoped their symptoms could have been acknowledged and not simply dismissed or perceived as an “inconsequential outcome” compared to their cancer [4, 50, 51, 55, 61].

I mentioned it [‘I have unbelievable pain with penetration’] to one of my […], and the answer was ‘if intercourse is a problem there’re other ways of pleasuring each other. (Sousa et al. [50], GA,Post,018)

Another study examining clinician-patient interactions suggested potential for more empathy and more thorough symptom assessment [61].

##### Expression of a desire for more support

Several studies reported cancer survivors’ need for extra support in managing their symptoms, especially amongst those who received no, or ineffective, treatment. In the study by Peate et al. [21] that included a community sample of breast cancer survivors, 53% needed additional support for sexual symptoms, and 60% for menopausal symptoms. In addition to this, six qualitative studies [4, 47, 49–51, 56] offered nuanced perspectives on cancer survivors’ support needs.

Four qualitative studies [47, 49, 51, 56] revealed that some cancer survivors felt uncertain about where to seek assistance, expressing desire for guidance in accessing support. For some cancer survivors, regular baseline and follow-up assessments during visits could help track symptom progression more effectively [4, 50], reducing reliance on self-recall [4].

But I don’t think anyone’s actually said, “Okay the last time you were having really bad hot flushes, this time they’re not so bad. I don’t think anyone’s ever said that. (Cruickshank and Hume [4], patient 1)

For cancer survivors experiencing genitourinary symptoms having a formal assessment process during follow-up, including physical examinations and consistent symptom monitoring could improve the care [50].

#### Theme 3: Information needs

Fourteen studies identified gaps in information provision (i.e. information needs), categorised into three subthemes: inadequate information about menopause pre-cancer treatment, insufficient guidance on symptom management, and reliance on diverse information sources (Table 3). Quantitative findings on information needs were limited, with only two contributing to the subtheme of inadequate information about menopause pre-cancer treatment.

##### Inadequate information about menopause before treatment

Two studies revealed significant gaps in providing menopause-related information to cancer survivors as a treatment side effect [53, 62]. A study of 346 young adult cancer survivors of diverse cancer types found that 60% received no information about their risk of early menopause before treatment [53]. Another study of 163 young breast cancer survivors showed that 27% and 32.5% received no information about secondary menopause associated with hormonal therapy and chemotherapy, respectively. Moreover, among those who did receive information, 18.5% were unsatisfied with hormonal therapy-related menopause information, and 24.5% with chemotherapy-related information [62].

Qualitative studies with similar cohorts [4, 48, 51, 52], including cancer survivors attending a survivorship clinic [48, 51], found that while some were informed about the potential effect of their treatment on their menstrual cycles, they struggled to understand its implications [4, 52] or the long-term consequences [48, 51]. Some cancer survivors reported difficulty distinguishing chemotherapy-related side effects from their menopausal symptoms, and whether their symptoms were caused by the treatment [4, 49, 51].

I didn’t know I was having menopausal symptoms... I was just talking to a friend…I said, “Well, it’s bearable…” And she goes, oh, I’ve got all that.”…made me realise that what I was experiencing wasn’t just solely because I was going through chemo…. (Balneaves et al. [51])

Many cancer survivors expected their HCPs to have informed them about menopause or provided more information [50, 56, 57]. Some felt their providers were uncomfortable discussing sexual and reproductive health topics during consultations [57]. Five studies showed where additional information could be useful to cancer survivors, including those about menopause as treatment side effect [54, 55, 57], its impact on their lives [47], and coping strategies [58].

It should be openly discussed by a treater [...] so we don’t feel, ‘hold on a second what’s happening in here?’… maybe say ‘look, ok it will all happen [...]maybe if you do this, it could help you. (Sousa et al. [50], GT,Pre,01)

##### Insufficient guidance on menopause symptom management

Five studies identified the opportunity for HCPs to expand their guidance for cancer survivors in managing menopausal symptoms [48, 50, 51, 54, 57]. Two studies reported cancer survivors’ concerns about the scarcity of, and barriers to receiving, information on managing their symptoms [48, 51].

I contacted the hospital myself because it was not endurable... They really should have informed and guided me on how to deal with that. (Sparidaens et al. [48], Q10,P17)

Cancer survivors who reported not to have received training [54], or information about treatments expressed that they could have benefited from such information had their HCPs shared with them [54, 56]. In the Sousa et al. [50] study, some cancer survivors were unaware of the treatment options for genitourinary symptoms, hence left their symptoms unmanaged.

##### Reliance on diverse sources for information

Across seven qualitative studies, cancer survivors sought information from diverse sources (Table 3) [4, 47–51, 57]. Many cancer survivors would have preferred HCPs as their primary source of information [47, 48, 50, 51, 57]; however, some reported that guidance was not proactively offered [50], some faced barriers in accessing available resources [4], and their needs were frequently unmet [57].

I suppose the breast care nurses would have been my chosen route…, I’m not actually sure that there’s enough opportunity to talk… (Cruickshank and Hume [4], patient 5)

When HCPs’ information was lacking, some women sought alternate sources including internet and social media [4, 47, 48, 50, 51, 57], electronic or printed materials [50, 51, 57], family and friends [4, 47, 49, 57], and other cancer survivors and support groups [47, 51, 57]. While some found these sources helpful [47], others questioned the credibility of the information [50, 51, 57].

I didn’t really know who to contact…my family gave various pieces of advice based on their personal experience. I suppose it was a bit more helpful (Singh et al. [49], P4)

## Discussion

This review showed that cancer survivors experiencing menopausal symptoms have significant unmet needs for managing their symptoms. The review included 20 studies made up of 3176 cancer survivors aged 18–97 years. All studies were conducted in high-(UK, Canada, Sweden, Australia, USA, The Netherlands) and middle-(Iran and Mexico) income countries. This uneven geographical distribution means that the findings may not represent the experiences of cancer survivors worldwide. Menopausal symptoms are subjective and understanding cancer survivors’ experiences and needs require in-depth personal accounts. This likely explains the predominance of qualitative over quantitative studies in this review. However, the quantitative findings aligned with and expanded upon the qualitative data, by uncovering unmet needs not assessed in the qualitative studies including cancer survivors untreated or found treatment ineffective.

The proportion of women reporting unmet needs varied widely across studies [14, 46, 59, 60], partly due to difference in study design and lack of validated menopause-specific unmet need assessment tool. While existing tools like CaSUN [66] and SCNS-SF34 [67] assesses menopausal symptoms unmet needs, none contain items on specific domains of needs (e.g. information, treatment). Thus, the need to develop a measure to be used for research and routine follow-up care. Most studies focused on breast cancer survivors, possibly due to their high prevalence and frequent experience of early menopause due to chemo- and hormonal therapies. However, the review showed that cancer survivors of other cancer types [49, 53, 58] shared similar unmet needs. Future studies should include cancer survivors of diverse cancer types, including rare cancers, to develop inclusive and effective strategies for all cancer survivors. Three key unmet needs were identified: for better menopause treatment [14, 15, 46, 56, 59, 60], support [4, 14, 46–51, 55–57, 60, 61], and information [4, 47–52, 54–58, 62].

### Treatment needs

Unmet treatment needs are a significant concern—26–80% of cancer survivors were untreated or self-managing their symptoms [14, 46, 60]. The difference observed could partly arise from variability in cultural, socioeconomic, and healthcare factors across the countries where the studies were conducted. Among the treated, effectiveness varied widely (15–76% found it ineffective), likely due to different interventions received. While Fenlon et al. [46] reported 15% ineffectiveness for CBT versus 49.2% and 76% for acupuncture and medical treatments [44] respectively, Peate et al. [14] found 34% completely ineffective treatments (type unspecified). Access (or lack thereof) to specialised menopause clinics may contribute to this heterogeneity. These clinics have shown effectiveness, with 83% of women reporting benefits [60], including improvements in hot flushes, sexual interest, and sleep [68]. Peate et al. [21] reported higher specialist access (3.8% vs 1.5% in Fenlon et al. [46]) and greater treatment effectiveness. The desire for more treatment was high—26–68% [59, 60] for vasomotor and 27–37% for genitourinary symptoms [59]. These findings not only demonstrate an urgent need for better management strategies but also showed symptom-specific treatment needs. The National Institute for Health and Care Excellence (NICE) guideline suggests involving menopausal women in decision-making while assessing and managing their symptoms individually [69].

### Support needs

This speaks to the need for better HCPs support; however, these findings primarily reflect breast cancer and gestational trophoblastic neoplasia [49] survivors’ needs. Global efforts to improve supportive care needs to address long-term effects are ongoing. The American Cancer Society and American Society of Clinical Oncology recommend monitoring cancer survivors for adverse effects that may negatively impact their quality of life [70]. In addition, the NICE [69] guideline addresses aspects of managing menopause in cancer survivors. However, this review suggests their implementation in clinical practice could be improved. Although participants expressed a need for symptom assessment tool, it is worth noting that several validated instruments do exist, such as the Hot Flash Related Daily Interference Scale and Day-to-Day Impact of Vaginal Aging [71, 72]. Specialised menopause clinics for cancer survivors assesses symptoms and offer relevant information [68]. Also, studies have shown that patient-reported outcomes [73] and web-based symptom monitoring [74] in cancer care can improve symptom management and QoL. Adopting similar strategies in primary care could address patient needs.

### Information needs

The final overarching finding concerned information needs. Cancer survivors from both middle-[52, 54] and high-[4, 47–51, 55–58] income countries reported unmet need for information about cancer treatment-induced menopause and symptom management. This demonstrates that these needs transcend economic contexts. While some HCPs may be unaware of existing resources, priorities of both providers and cancer survivors during treatment may contribute to this need. Timely information provision, while considering each woman’s needs, readiness, and processing ability [49] could increase their overall satisfaction. Cancer survivors relied on various sources including HCPs, yet their information needs were unmet. Reluctance to provide such information may arise from limited training among HCP [75]. Undoubtedly, workshops and webinars from menopause societies could enhance HCPs’ knowledge, but integrating menopause training into medical school curricula provide solid foundation for all future providers. In addition, directing cancer survivors to alternative sources like cancer charities and peer support groups could supplement information delivery. Providing evidence-based information, as recommended by the British Menopause Society guideline, ensures that cancer survivors are well-informed and their mental well-being are safeguard [76].

### Opportunities to improve information, support, and treatment needs

Table 4 outlines an approach to address cancer survivors’ unmet needs throughout the cancer journey. During treatment planning, HCPs could provide information and/or direct cancer survivors to resources about menopause after cancer treatment. In the active treatment, HCP could assess, discuss treatment choices, and manage emerging menopausal symptoms. Post-treatment and during follow-ups, HCP could regularly assess symptom, manage or refer to a level of care based on symptom burden/impact. Throughout this stage, HCP could also direct cancer survivors to cancer charities, NGOs, and peer groups to provide ongoing support, information, and resources.

**Table 4:**
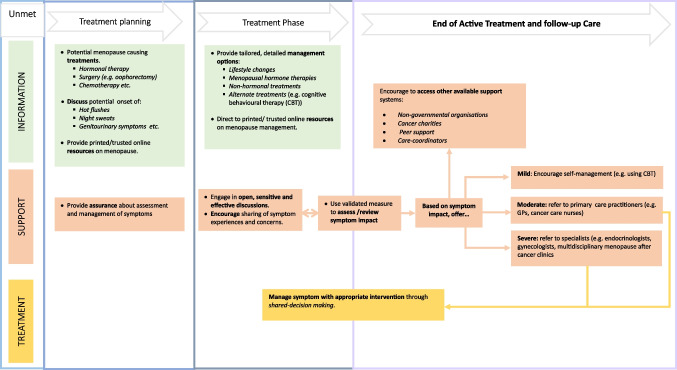
Opportunities for HCP to improve information, support, and treatment needs in cancer survivors’ journey

### Operationalising findings within survivorship and supportive care services

This systematic reviewed identified cancer survivors’ needs for treatment, information, and support for managing menopausal symptoms after cancer, with important implications for design and delivery of survivorship and supportive care services. Firstly, routine screening for menopausal symptoms and any related unmet needs should be embedded in survivorship and supportive care plans to ensure that all cancer survivors experiencing menopause after cancer receive appropriate support. This could be facilitated through the implementation of PROMs, unmet needs assessment instruments, and clear referral pathways. Secondly, clinical care should be more tailored and more patient-centred. Stratified- or stepped-care models offer a practical approach for delivering menopause after cancer care by enabling screening and triaging of survivors to appropriate levels of care, including self‑management, GP/nurse‑led care, or specialist-led services. Such models could be integrated within existing multidisciplinary survivorship care frameworks. Finally, cancer survivors and HCPs across disciplines should be actively involved in the co-design of tailored, evidence‑based information resources. These may include written materials (e.g. flyers, booklets, pamphlets) or online platforms (e.g. website or mobile apps), ensuring that resources are relevant, accessible, and responsive to survivors’ information needs regarding menopause after cancer.

## Clinical implications and future research

These findings suggest ways HCPs could improve clinical practice to address the identified unmet needs. HCP could be more receptive and provide an environment where cancer survivors feel heard, and their symptoms managed. Unmet needs, if addressed, could improve overall QoL and mental well-being of cancer survivors. Furthermore, the findings could inform supportive care strategies tailored to these needs. Most studies were in breast cancer survivors and few primarily investigated menopausal symptoms unmet needs. Future studies should explore menopause-related needs and beyond breast cancer survivors to direct future supportive care practices.

## Strength and limitations

This review is the first to examine the unmet needs of cancer survivors experiencing menopausal symptoms, addressing a critical literature gap. This study reports on both researchers’ perspectives and patient’s voices about their experiences and needs, hence allowing for real-world applicability of the finding. Triangulating qualitative and quantitative findings allowed for themes and subthemes to be interpreted from multiple perspectives. Also, adhering to PRISMA guideline allowed for methodological rigour in the search, screening, and selection processes. Additionally, it was comprehensive in scope (not limited to one cancer type). However, generalisability is limited to the inclusion criteria and participants. Finally, as studies were mostly from Western countries, our findings may not reflect geographical and socio-economic inclusiveness.

## Conclusion

Most cancer survivors have unmet needs regarding menopausal symptoms after cancer treatment management. The review identified three main needs across treatment, support, and information. To meet these needs, we suggest that HCPs could give and/or direct cancer survivors to information resources about post-treatment menopause, and available interventions for management. This guidance could be offered throughout the cancer journey, from active treatment planning to follow-up care. We recommend that HCPs routinely assess, treat, and/or refer cancer survivors with menopausal symptoms to other specialists during and after cancer treatment. We also suggest solutions such as online resources and a stepped care model to manage menopause in cancer survivors. This approach could improve access to self-help resources, primary care, and specialists. Also, expanding menopause after cancer clinics could help to situate menopause care in survivorship pathways and support more equitable access to evidence-based services. Lastly, we recommend integration of menopause training in medical school curricula and allocation of resources for specialised training to enable all HCPs, including future providers, to have the requisite skills to manage menopausal symptoms in cancer. The review could provide a foundation to offering care for cancer survivors with menopause after cancer.

## Data Availability

No datasets were generated or analysed during the current study.

## Appendix 1 Search strategy-ovid MEDLINE(R) ALL, embase classic + embase, psychoINFO, and CINAHL

Two strategies were employed: four electronic databases (Ovid MEDLINE(R) ALL, Embase Classic + Embase, APA PsycINFO, and CINAHL) were searched using MeSH terms and keywords.

Search strategy-Ovid MEDLINE(R) ALL, Embase Classic + Embase, PsychoINFO, and CINAHL.

**Table:**

	**Ovid MEDLINE(R) ALL**	
S5	**Limit S4 to (English language and female and human and (2013–2025))**	**2481**
S4	S1 AND S2 AND S3	6813
S3	exp Menopause/or exp Menopause, Premature/or Menopause.mp. or “Menopause Premature”.mp. or exp Climacteric/or Climacteric.mp. or Climacteric symptom.mp. or exp Postmenopause/or Postmenopaus*.mp. or Climacteric syndrome.mp. or Menopaus*.mp. or Menopausal symptom*.mp. or “Early Menopause”.mp. or “Night sweat*”.mp. or “Vasomotor symptom*”.mp. or exp Hot Flashes/or “Hot Flash*”.mp. or “Hot flush*”.mp. or “hotflash*”.mp. or “hotflush*”.mp. or Symptom*.mp	1,718,943
S2	exp Cancer Survivors/or “cancer survivor*”.mp. or exp Survivors/or Survivor*.mp. or Survivor*.mp. or “cancer patient*”.mp. or exp Carcinoma/or Carcinoma.mp. or exp Neoplasms/or Neoplasm*.mp. or cancer remission.mp. or “long-term survivor*”.mp. or exp Breast Neoplasms/or Breast Neoplasm*.mp. or “Breast Cancer*”.mp	4,549,892
S1	exp Needs Assessment/or Needs Assess*.mp. or “unmet need*”.mp. or “unmet demand*”.mp. or “symptom manag*”.mp. or “Supportive need*”.mp. or “Information need*”.mp. or “Decision Support*”.mp. or “healthcare need*”.mp. or “supportive care need*”.mp. or ((assess* or support* or information* or evaluati*) adj3 need*).mp	261,557
	**Embase Classic + Embase**	
	**Search terms**	
S5	**Limit S4 to (English language and female and human and (2013–2025)) NOT conference abstracts**	**3520**
S4	S1 AND S2 AND S3	14,026
S3	exp Menopause/or exp Menopause, Premature/or Menopause.mp. or “Menopause Premature”.mp. or exp Climacteric/or Climacteric.mp. or Climacteric symptom.mp. or exp Postmenopause/or Postmenopaus*.mp. or Climacteric syndrome.mp. or Menopaus*.mp. or Menopausal symptom*.mp. or “Early Menopause”.mp. or “Night sweat*”.mp. or “Vasomotor symptom*”.mp. or exp Hot Flashes/or “Hot Flash*”.mp. or “Hot flush*”.mp. or “hotflash*”.mp. or “hotflush*”.mp. or Symptom*.mp	2,834,260
S2	exp Cancer Survivors/or “cancer survivor*”.mp. or exp Survivors/or Survivor*.mp. or Survivor*.mp. or “cancer patient*”.mp. or exp Carcinoma/or Carcinoma.mp. or exp Neoplasms/or Neoplasm*.mp. or cancer remission.mp. or “long-term survivor*”.mp. or exp Breast Neoplasms/or Breast Neoplasm*.mp. or “Breast Cancer*”.mp	7,160,797
S1	exp Needs Assessment/or Needs Assess*.mp. or “unmet need*”.mp. or “unmet demand*”.mp. or “symptom manag*”.mp. or “Supportive need*”.mp. or “Information need*”.mp. or “Decision Support*”.mp. or “healthcare need*”.mp. or “supportive care need*”.mp. or ((assess* or support* or information* or evaluati*) adj3 need*).mp	338,003
	**PsychoINFO**	
	**Search terms**	
S5	**Limit S4 to (English language and female and (2013–2025))**	**868**
S4	S1 AND S2 AND S3	1815
S3	exp Menopause/or exp Menopause, Premature/or Menopause.mp. or “Menopause Premature”.mp. or exp Climacteric/or Climacteric.mp. or Climacteric symptom.mp. or exp Postmenopause/or Postmenopaus*.mp. or Climacteric syndrome.mp. or Menopaus*.mp. or Menopausal symptom*.mp. or “Early Menopause”.mp. or “Night sweat*”.mp. or “Vasomotor symptom*”.mp. or exp Hot Flashes/or “Hot Flash*”.mp. or “Hot flush*”.mp. or “hotflash*”.mp. or “hotflush*”.mp. or Symptom*.mp	482,135
S2	exp Cancer Survivors/or “cancer survivor*”.mp. or exp Survivors/or Survivor*.mp. or Survivor*.mp. or “cancer patient*”.mp. or exp Carcinoma/or Carcinoma.mp. or exp Neoplasms/or Neoplasm*.mp. or cancer remission.mp. or “long-term survivor*”.mp. or exp Breast Neoplasms/or Breast Neoplasm*.mp. or “Breast Cancer*”.mp	113,887
S1	exp Needs Assessment/or Needs Assess*.mp. or “unmet need*”.mp. or “unmet demand*”.mp. or “symptom manag*”.mp. or “Supportive need*”.mp. or “Information need*”.mp. or “Decision Support*”.mp. or “healthcare need*”.mp. or “supportive care need*”.mp. or ((assess* or support* or information* or evaluati*) adj3 need*).mp	82,955
	**CINAHL**	
	**Search terms**	
	Limit S4 to (English and female (2013–2025))	**1383**
S4	S1 AND S2 AND S3	4793
S3	((MH “Menopause + ”) OR TI menopau* OR AB menopau*) OR TI (premature menopause or early menopause or induced menopause) OR AB (premature menopause or early menopause or induced menopause) OR ((MH “Climacteric + ”) OR TI climacteric OR AB climacteric OR TI “Climacteric symptom*” OR AB “Climacteric symptom*” OR TI “Climacteric syndrome” OR AB “Climacteric syndrome”) OR TI “Night sweat*” OR AB “Night sweat*” OR TI “Hot flush*” AB “Hot flush*” OR ((MH “Hot Flashes”) OR TI “Hot Flash*” OR AB “Hot Flash*” OR TI “hotflash*” OR AB “hotflash*”) OR (MH symptom* OR TI symptom* OR AB symptom*))	443,092
S2	(((MH “Cancer Survivors”) OR TI (cancer survivors or survivors of cancer or cancer survivorship or survivorship or after cancer treatment OR AB (cancer survivors or survivors of cancer or cancer survivorship or survivorship or after cancer treatment)) OR ((MH “Cancer Patients”) OR TI (cancer patients or oncology patients or patients with cancer) OR AB (cancer patients or oncology patients or patients with cancer)) OR ((MH “Carcinoma + ”) OR TI Carcinoma AB Carcinoma) OR ((MH “Neoplasms + ”) OR TI Neoplasms OR AB Neoplasms) OR (TI cancer remission OR AB cancer remission) OR (TI “long-term survivor* OR AB “long-term survivor*) OR ((“MH breast neoplasms OR TI (breast cancer or breast neoplasm or breast carcinoma or breast tumor) OR AB (breast cancer or breast neoplasm or breast carcinoma or breast tumor)	833,680
S1	((MH “Needs Assessment”) OR TI Needs Assessment OR AB Needs Assessment) OR (TI “Unmet need*” OR AB “Unmet need*”) OR (TI “unmet demand*” OR AB “unmet demand*”) OR (TI symptom management OR AB symptom management) OR (TI supportive care needs OR AB supportive care needs) OR (TI information need OR AB information need) OR (TI decision support OR AB decision support) OR (TI healthcare needs OR AB healthcare needs)	106,105

## Appendix 2 Critical appraisal of included quantitative studies using JBI checklist (*n* = 7)

The Joanna Briggs Institute (JBI) checklists were used for critical appraisal for qualitative studies; scores ≤ 49%, 50–69%, and ≥ 70% “yes” were considered low, moderate, and high quality, respectively. Four of the seven studies were considered high quality and three as moderate.

Critical appraisal of included quantitative studies using JBI checklist (*n* = 7).

**Table:**

JBI questions	Author, year published						
	**Cole et al. 2022 **[60]	**Capelan et al. 2017 **[15]	**Fenlon et al. 2022 **[46]	**Miyashita et al. 2015 **[62]	**Palmer et al. 2016 **[59]	**Peate et al. 2020 **[14]	**Benedict et al. 2016 **[53]
Was the sample frame appropriate to address the target population?	Yes	Yes	Yes	Yes	Yes	Yes	Yes
Were study participants sampled in an appropriate way?	Yes	Yes	No	No	No	Yes	Yes
Was the sample size adequate?	Yes	Yes	No	No	No	Yes	Yes
Were the study subjects and the setting described in detail?	Yes	Yes	Yes	Yes	Yes	Yes	Yes
Was the data analysis conducted with sufficient coverage of the identified sample?	No	No	Yes	Yes	No	No	No
Were valid methods used for the identification of the condition?	Yes	Yes	Yes	Yes	Yes	Yes	Yes
Was the condition measured in a standard, reliable way for all participants?	Yes	Yes	Yes	Yes	Yes	Yes	Yes
Was there appropriate statistical analysis?	Yes	Yes	Yes	Yes	Yes	Yes	Yes
Was the response rate adequate, and if not, was the low response rate managed appropriately?	Yes	Yes	No	No	Yes	Yes	Yes
Quality rating = (Total no. of JBI items (Yes))	8/9	8/9	6/9	6/9	6/9	8/9	8/9

## Appendix 3 Critical appraisal of included qualitative studies using JBI checklist (*n* = 13)

Critical appraisal used Joanna Briggs Institute (JBI) checklists for quantitative studies with “yes” or “no” responses. Studies scoring ≤ 49%, 50–69%, and ≥ 70% “yes” were considered low, moderate, and high quality, respectively. All were high quality.

**Table:**

JBI questions	Author, year published												
	**Cruickshank and Hume (2014) **[4]	**Balneaves et al. 2016 **[51]	**Johnsson et al. 2023 **[47]	**Sousa et al. 2017 **[50]	**Limbacher et al. 2022 **[61]	**Black et al. 2020 **[57]	**Sparidaens et al. 2022 **[48]	**Lopez et al. 2019 **[58]	**Moreno et al. 2023 **[56]	**Khajoei et al. 2024 **[59]	**Ko et al. 2023 **[55]	**Singh et al. 2024 **[49]	**Villarreal‐Garza et al. 2019 **[52]
Is there congruity between the…													
…stated philosophical perspective and the research methodology?	Yes	Yes	Yes	No	No	Yes	Yes	No	Yes	Yes	Yes	Yes	Yes
…research methodology and the research question or objectives?	Yes	Yes	Yes	Yes	Yes	Yes	Yes	Yes	Yes	Yes	Yes	Yes	Yes
…research methodology and the methods used to collect data?	Yes	Yes	Yes	Yes	Yes	Yes	Yes	Yes	Yes	Yes	Yes	Yes	Yes
…research methodology and the representation and analysis of data?	Yes	Yes	Yes	Yes	Yes	Yes	Yes	Yes	Yes	Yes	Yes	Yes	Yes
…research methodology and the interpretation of results?	Yes	Yes	Yes	Yes	Yes	Yes	Yes	Yes	Yes	Yes	Yes	Yes	Yes
Is there a statement locating the researcher culturally or theoretically?	No	No	No	Yes	Yes	Yes	Yes	Yes	Yes	No	Yes	Yes	Yes
Is the influence of the researcher on the research, and vice- versa, addressed?	No	No	Yes	Yes	Yes	Yes	Yes	Yes	No	No	No	No	Yes
Are participants, and their voices, adequately represented?	Yes	Yes	Yes	Yes	Yes	Yes	Yes	Yes	Yes	Yes	Yes	Yes	Yes
Is the research ethical according to current criteria or, for recent studies, and is there evidence of ethical approval by an appropriate body?	Yes	Yes	Yes	Yes	Yes	Yes	Yes	Yes	Yes	Yes	Yes	Yes	Yes
Do the conclusions drawn in the research report flow from the analysis, or interpretation, of the data?	Yes	Yes	Yes	Yes	Yes	Yes	Yes	Yes	Yes	Yes	Yes	Yes	Yes
Quality rating = (Total no. of JBI items (Yes))	8/10	8/10	9/10	9/10	9/10	10/10	10/10	9/10	9/10	8/10	9/10	9/10	10/10
